# The Pathways to Create Containers for Bacteriophage Delivery

**DOI:** 10.3390/polym14030613

**Published:** 2022-02-04

**Authors:** Egor V. Musin, Aleksandr L. Kim, Alexey V. Dubrovskii, Elena V. Ariskina, Ekaterina B. Kudryashova, Sergey A. Tikhonenko

**Affiliations:** 1 Institute of Theoretical and Experimental Biophysics, Russian Academy of Science, Institutskaya St., 3, 142290 Puschino, Moscow Region, Russia; eglork@gmail.com (E.V.M.); kimerzent@gmail.com (A.L.K.); dav198@mail.ru (A.V.D.); 2 All-Russian Collection of Microorganisms (VKM), G. K. Skryabin Institute of Biochemistry and Physiology of Microorganisms, Federal Research Center, Pushchino Scientific Center for Biological Research of the Russian Academy of Sciences, Prospect Nauki 5, 142290 Pushchino, Moscow Region, Russia; katryn@ibpm.pushchino.ru (E.B.K.); lena@ibpm.pushchino.ru (E.V.A.)

**Keywords:** microcapsules, bacteriophage, *E. coli*, CaCO_3_, encapsulation, polyarginine, dextran sulfate

## Abstract

Antimicrobial resistance is a global public health threat. One of the possible ways to solve this problem is phage therapy, but the instability of bacteriophages hinders the development of this approach. A bacteriophage delivery system that stabilizes the phage is one of the possible solutions to this problem. This study is dedicated to exploring methods to create encapsulated forms of bacteriophages for delivery. We studied the effect of proteolytic enzymes on the destruction of the polyelectrolyte microcapsule shell and revealed that protease from *Streptomyces griseus* was able to destroy the membrane of the microcapsule (dextran sulfate/polyarginine)_3_ ((DS/PArg)_3_). In addition, the protease decreased the activity of the bacteriophage in the second hour of incubation, and the phage lost activity after 16 h. It was found that a medium with pH 9.02 did not affect the survival of the bacteriophage or *E. coli*. The bacteriophages were encapsulated into polyelectrolyte microcapsules (DS/PArg)_3_. It was established that it is impossible to use microcapsules as a means of delivering bacteriophages since the bacteriophages are inactivated. When bacteriophages were included inside a CaCO_3_ core, it was demonstrated that the phage retained activity before and after the dissolution of the CaCO_3_ particle. From the results of this study, we recommend using CaCO_3_ microparticles as a container for bacteriophage delivery through the acidic stomach barrier.

## 1. Introduction

Antimicrobial resistance occurs when bacteria of a certain type acquire the ability to protect themselves from antibiotics, which normally effectively disrupt their vital activity. It significantly complicates the treatment of infectious diseases that they cause. The Centers for Disease Control and Prevention reported that in the United States, the number of reported deaths due to antibiotic-resistant infections reaches 35,000 per year [1]. It is necessary to develop not only new antibiotics but also new methods of treating bacterial infections to slow down the emergence of resistant bacterial strains [2].

One of the possible ways to solve this problem is bacteriophages. Bacteriophages are viruses that can selectively infect the bacterial cells of one strain or antigenically homologous strains of the same species or genus. This infection is followed by lysis (after intracellular replication, except for temperate or chronic phages) of the bacterial host cell [3] and does not threaten eukaryotic cells. Phages are used as natural antimicrobial agents to fight bacterial infections in humans, animals and crops [4,5,6,7,8]. Bacteriophages are used in phage therapy. This is a direction in medicine that allows the treatment of bacterial infections by ingestion or local application of a specific polyvalent phage [9]. Phage therapy is rapidly developing around the world and has great prospects; however, the development of this strategy is hindered by the instability of the bacteriophage [10]. Thus, it is necessary to develop effective delivery systems capable of protecting the bacteriophage from the external environment. One of the ways to create this system is to encapsulate bacteriophages.

Both organic and inorganic particles can be used as containers for bacteriophages. Among inorganic particles, porous calcium phosphate particles are most often used as a carrier of bacteriophages [11,12]. J.C. Hornez et al. loaded 1 µm calcium phosphate beads and suggested using microspheres as a matrix for a local drug delivery system to prevent and cure infections associated with bone implants [12].

Alginate is one of the most commonly used organic materials for bacteriophage encapsulation [13]. Alginate microcapsules possess mucoadhesive properties that allow them to entrap molecules higher than 10,000 Da [14]. However, this type of encapsulation is not suitable for all bacteriophages; therefore, it is necessary to study new methods for encapsulating phages [15]. Chitosan particles are the second most often used organic microparticles for bacteriophage delivery [14,16,17]. In addition to chitosan, other polymer compounds are used to create microcapsules: poly(ethylene oxide)/cellulose diacetate fibers [18], poly(acryl starch) and poly(lactide-co-glycolide) [19]. For example, Jamaledin R et al. used microparticles (MPs) made of poly(lactic-co-glycolic acid) (PLGA) to encapsulate fd bacteriophages, and it was revealed that the encapsulated bacteriophages were stable and retained their immunogenic properties. In addition, the combination of alginate with carrageenan, chitosan and whey protein was used for bacteriophage encapsulation, and these phages remained viable even when subjected to pH 2.5 for 2 h [20]. Thus, the use of polyelectrolytes is a promising direction for the encapsulation of bacteriophages [21].

Polyelectrolyte nano- and microcapsules (PMCs) are objects of a new rapidly developing field—polymer nanotechnology. They are made by alternately layering oppositely charged polyelectrolytes on dispersed nano- or microsized particles, with their subsequent destruction and removal [22,23,24]. Polyelectrolyte capsules are used as sensor systems for the determination of low-molecular-weight substances in multicomponent media [25,26,27,28,29], as delivery systems for biologically active components to cells and tissues [30,31,32,33] and as materials in the creation of prolonged-release medicines [34,35,36]. In this context, many works have been devoted to biocompatibility, biodistribution, biodegradation and clearance from the body [30,37]. In particular, S. De Koker et al. demonstrated that most of the polyelectrolyte microcapsules are internalized by the cells and start to degrade [38]. However, works devoted to the encapsulation of bacteriophages in polyelectrolyte microcapsules could not be found.

A specific destruction system of the PMC shell requires the successful delivery of bacteriophages. At the moment, there are several ways to decapsulate substances from polyelectrolyte microcapsules: using light (by destroying photosensitive polymers) [39], using a constant magnetic field (due to the presence of a magnetic core particle in the PMC shell) [40], with the help of proteolytic enzymes (in this case, the PMC shell consists of biodegradable polyelectrolytes) [41], by changing the pH of the medium [42], with the help of bacterial spores (due to the germination of bacterial spores on a nutrient medium) [43] and other methods.

It is necessary to study the effect of polyelectrolytes on phage activity to encapsulate bacteriophages in PMCs while maintaining their activity. At the moment, this problem remains under-researched and is discussed in only a few publications, which did not study the effect of polyanions on the activity of bacteriophages. Few studies have demonstrated a decrease in phage activity in the presence of polyamino acids. In particular, most of the works are devoted to the antiviral and antibacterial properties of polylysine [44,45]. For example, S. Shima et al. [46] showed that the cationic polyelectrolyte poly-L-lysine caused a 2.5-fold reduction in the activity of the bacteriophage at a polymer concentration of 10 μg/mL; at a concentration of 1 mg/mL, complete inactivation of the T4 bacteriophage was observed. In addition, polyelectrolytes based on amino acids, such as poly-y-methyl-γ, L-glutamate and poly-N-(p-aminoethyl) glutamine, were synthesized, and they also reduced the activity of bacteriophages T4 and T5 [47].

The aim of this work was to develop encapsulated forms of bacteriophages for delivery into the small intestine.

## 2. Materials and Methods

Poly-L-lysine hydrobromide (PL, MW 70,000), poly-L-arginine hydrochloride (PArg, MW 15,000–70,000) and sodium dextran sulfate (DS, MW 50,000) were from Sigma (Neustadt, Germany); bacteriophage of Proteus vulgaris and *Escherichia coli* (Microgen NPO AO, Microgen, Moscow, Russia), ethylenediaminetetraacetic acid (EDTA), calcium chloride (CaCl_2_·2H_2_O), sodium chloride and sodium carbonate were from Reahim (Ekaterinburg, Russia). Protease from *Streptomyces griseus* (Type VI No. P-5130) was obtained from Sigma (Neustadt, Germany).

### 2.1. Bacterial Strains and Their Cultivation

We used a daily culture of *Escherichia coli* K12. The strain was grown on LB agar medium in test tubes at 37 °C. A cell suspension was prepared in sterile distilled water, with a density of 15 × 108/mL (No. 5 according to McFarland).

### 2.2. Sowing

A 100 µL aliquot of the test suspension (encapsulated phage/native phage) and 100 µL *E. coli* K12 suspension were applied on the surface of LB agar medium/McConkey-GRM medium (Obolensk, Russia) on a Petri plate. After incubation for 2 days at 37 °C, we determined the number of plaques on the agar surface. The presence of a plaque on the agar surface is evidence of bacteriophage activity. The sowing of each sample was carried out in triplicate.

### 2.3. Preparation of CaCO_3_ Microspherolites

An equal volume of 0.33 M aqueous Na_2_CO_3_ solution was added to a 0.33 M aqueous solution of CaCl_2_ containing bacteriophages, and the mixture was vigorously stirred on a magnetic stirrer [48]. Stirring was continued for 30 s at 300 rpm, after which it was stopped, and the resulting suspension was maintained until the formed particles completely settled. The process of “maturation” of microspherolites was monitored using a light microscope. Then, the supernatant was decanted, and the precipitate was washed with water and used to obtain polyelectrolyte microcapsules (PMCs). The number of included bacteriophages was determined by adding the supernatant to the LB medium/McConkey-GRM medium (Obolensk, Russia) with *E. coli*, further calculating the number of plaques of supernatant and the number of plaques of initial bacteriophage concentration and determining the difference. The size of particles was controlled by light microscopy and determined in the program ImageJ. The number of formed microspherolites was determined in the Goryaev chamber (40 × 10^6^).

### 2.4. Preparation of Polyelectrolyte Microcapsules (PMCs)

**Figure 1:**
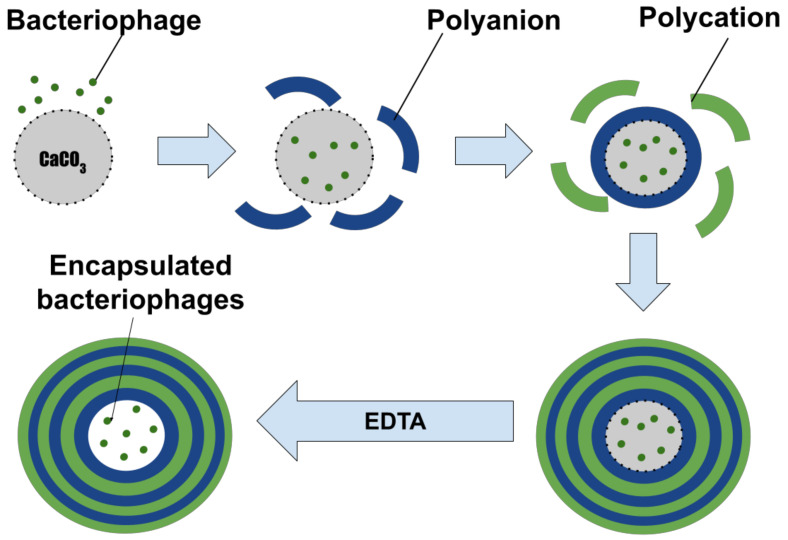
Scheme of polyelectrolyte microcapsule preparation.

PMCs were prepared under aseptic conditions using sterile solutions. Polyelectrolyte microcapsules were obtained by alternate adsorption of oppositely charged polyelectrolytes on a dispersed microparticle (microspherolite), followed by its dissolution (Figure 1) [12]. Alternative adsorption of DS and PArg on the surface of CaCO_3_ microspherolites was carried out in polyelectrolyte solutions with a concentration of 2 mg/mL containing a 0.5 M NaCl solution. After each stage of adsorption, it was washed three times with a solution of 0.5 M NaCl, which is necessary to remove unadsorbed polymer molecules. The particles were separated from the supernatant by centrifugation at 500 g. After six or seven layers were applied, the carbonate cores were dissolved in 0.2 M EDTA solution for 2 h. The resulting capsules were washed three times with water to remove core decay products. The resulting microparticles had a diameter of 4.5 ± 1 μm. The size of microcapsules was measured using the dynamic light scattering method on a Zetasizer Nano ZS device (Malvern, UK).

### 2.5. Phage Labeling

Phages were labeled with FITC. Phages (1 × 10^12^ pfu) were resuspended in 100 mL of a 0.3 M NaHCO_3_ (pH 8.6) solution containing 0.25 mg/mL FITC. The concentration of fluorochrome was determined spectrophotometrically (Varian Cary II; Varian, Palo Alto, CA, USA). 

### 2.6. Registration of the Bacteriophage Release from Polyelectrolyte Microcapsules and CaCO_3_ Microparticles

Bacteriophage release from polyelectrolyte microcapsules and CaCO_3_ microparticles was studied by fluorescence spectroscopy. Polyelectrolyte microcapsules or CaCO_3_ microparticles containing FITC-labeled bacteriophages were precipitated by centrifugation at 3000 rpm for 1 min. Further, the fluorescence of the supernatant was measured. Fluorescence spectra were recorded on an Infinite 200 Tecan instrument in a thermostated cuvette with an optical path length of 1 cm when excited with light at a wavelength of 273 nm.

### 2.7. Fluorescence Microscopy

Images were taken using an «Axiovert 200M Cell Observer» inverted fluorescence microscope with a high-speed camera (AxioCam HSM of Carl Zeiss Company; Jena, Germany) using 60×/1.4 oil objective. Diode laser 488 was used for excitation. Images were acquired at a resolution of 1388 × 1040 pixels.

## 3. Results

**Figure 2:**
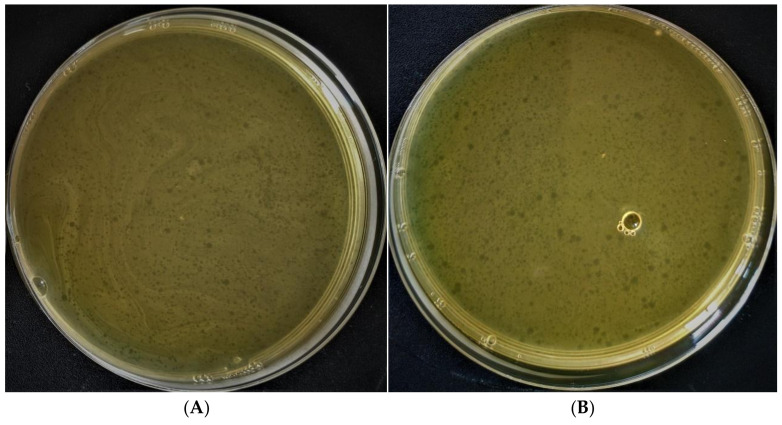
Influence of the alkaline medium on survival of bacteriophages. (**A**) Medium with pH 9.02; (**B**) medium with pH 5.5.

Previous research on the medium’s influence on phage stability [49,50] shows that some bacteriophages do not survive at alkaline pH values of the medium. The first stage of microcapsule formation is the creation of the CaCO_3_ core, which changes the pH of the medium to alkaline values. For this reason, we examined the effect of the medium with pH = 9.02 on phage activity, which makes it possible to simulate the conditions during the formation of the CaCO_3_ core at the first stage of microcapsule formation. Figure 2 shows that the number of plaques formed at pH = 9.02 (408 plaques) and 5.5 (426 plaques) did not differ significantly; that is, the high pH of the solution did not affect the survival of the bacteriophage. The results obtained coincide with the data of the article by M. K. Taj et al., in which the bacteriophage of Escherichia coli was stable in a pH range from 4 to 10 [51].

**Figure 3:**
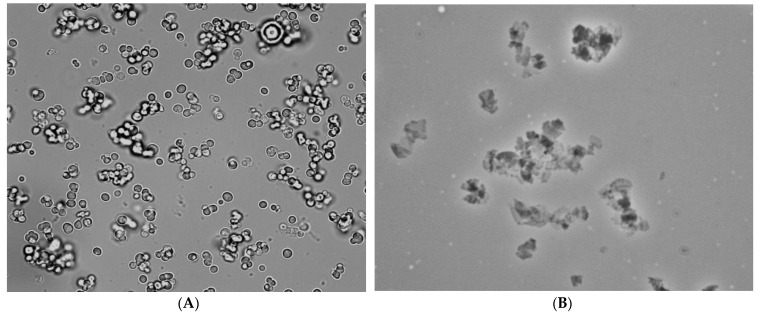
Degradation of the shell of polyelectrolyte microcapsules by proteinase from *Streptomyces griseus*. (**A**) PMCs before proteinase; (**B**) PMCs after proteinase.

The literature reports a narrow spectrum of proteolytic enzymes that break down the membrane of the PMC. In the work of A.M. Pavlov et al. [52], it was shown that biodegradable polyelectrolyte microcapsules are destroyed by proteolytic enzymes, in particular, trypsin, while Craig M. et al. used protease IV from *Pseudomonas aeruginosa* [41]. Therefore, the following proteinases were tested to select a proteinase capable of destroying the membrane of biodegradable PMCs (DS/PARg)_3_: trypsin, proteinase P from *Streptomyces griseus* (Sigma), trypsin from bovine pancreas, protease from a strain of B. subtilis and protease from *Streptomyces griseus* (SERVA). As a result, proteinase P from *Streptomyces griseus* (0.8 mg/mL) (manufactured by Sigma, Neustadt, Germany, and Serva, Heidelberg, Germany) was confirmed to be suitable for the destruction of PMCs (Figure 3) with incubation for 2 h at 37 °C, unlike other proteinases.

**Figure 4:**
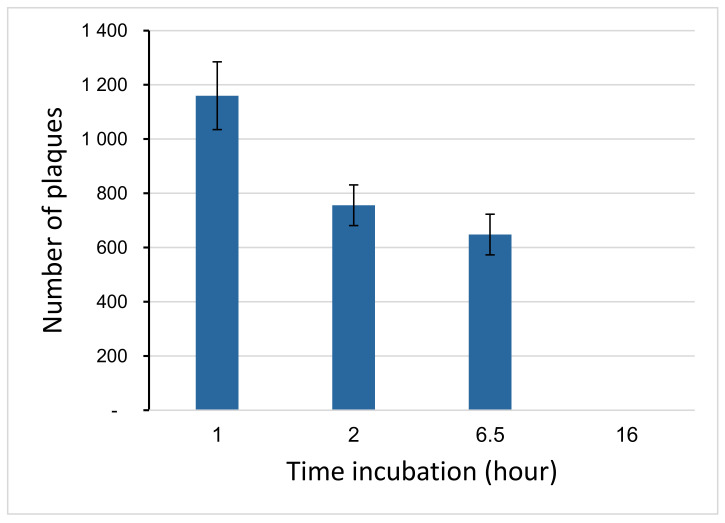
Survival of bacteriophage in proteolytic enzyme depending on the incubation time.

Previously, it was shown that proteolytic enzymes can affect the stability of a bacteriophage. In natural conditions, bacteria use proteases as protection against phages [53,54]. In this regard, we studied the effect of proteinase P from *Streptomyces griseus* on phage activity. Figure 4 shows decreases in phage viability in the second hour of incubation. Further, the viability of the bacteriophage was completely eliminated at 16 h of incubation. Thus, it can be concluded that the incubation of encapsulated bacteriophages in a proteolytic enzyme should not exceed 6.5 h.

**Figure 5:**
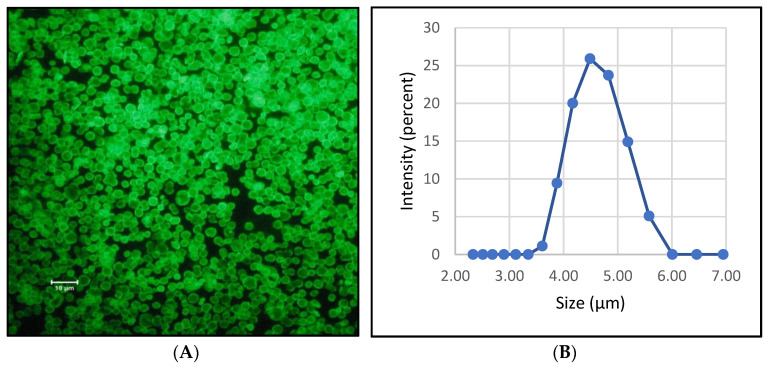
Image (**A**) and size distribution by intensity (**B**) of polyelectrolyte microcapsules with encapsulated bacteriophages. (**A**) Fluorescence microscopy image of PMCs with encapsulated FITC-labeled bacteriophages; (**B**) size distribution by intensity of PMCs with encapsulated bacteriophages.

Subsequently, the bacteriophages were encapsulated in biodegradable six-layer polyelectrolyte microcapsules. The composition of the shells of these PMCs was (DS/PArg)_3_ (Table 1). The results of fluorescence microscopy of polyelectrolyte microcapsules demonstrated the successful encapsulation of bacteriophages into PMCs (Figure 5A). The size distribution of PMCs was also measured, and the results are shown in Figure 5B. The average microcapsules size is 4.5 ± 1 µm.

**Figure 6:**
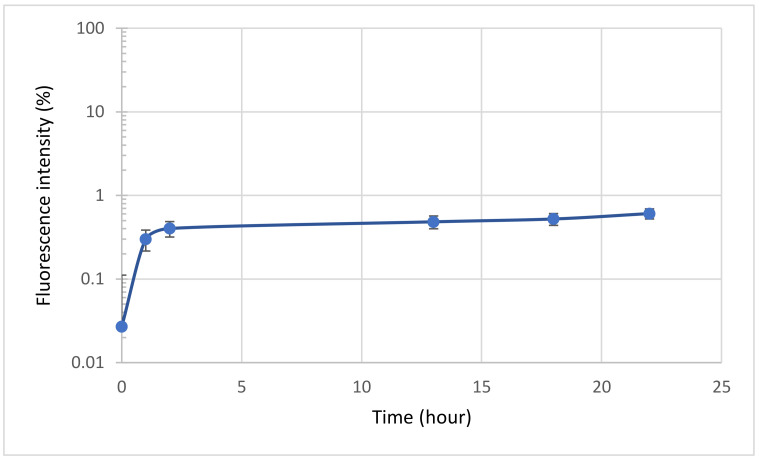
Bacteriophage release from polyelectrolyte microcapsules depending on the incubation time.

After encapsulating the bacteriophages, the amount of bacteriophage released from polyelectrolyte microcapsules depending on the incubation time was determined. To that end, FITC-labeled bacteriophages were encapsulated and incubated in an aqueous solution at 37 °C. Figure 6 shows that the release of the bacteriophage from the PMC is insignificant compared to the starting value (100%—the total encapsulated labeled bacteriophage). Thus, it can be concluded that the incubation of encapsulated bacteriophages in an aqueous solution at 37 °C does not affect phage release.

Subsequently, the survival of bacteriophages after the destruction of PMC shells was studied. As can be seen from Table 1, the encapsulated bacteriophages lost their activity after the destruction of the PMC shell. We put forward two hypotheses for the loss of phage activity: the envelope polyelectrolyte complex inhibited the phage activity, or the bacteriophages emerged from the capsule upon the destruction of the CaCO_3_ core [55].

We created two types of capsules to test these hypotheses. In the first type, the CaCO_3_ core was not destroyed at the last stage of the encapsulation of the bacteriophage; this will prevent the interaction of the PMC shell with the phage capsid. In the second type of capsules, the complex of the bacteriophage with the polyelectrolyte PDADMAC was encapsulated. In the work of Jabrane et al., the possibility of immobilizing the bacteriophage using PDADMAC while maintaining its activity on paper was shown [55]. In this way, we hypothesized that PDADMAC would allow the viral particle to be maintained inside the capsule after the destruction of the CaCO_3_ core. As a result, after the addition of capsules to the nutrient medium with the subsequent destruction of the PMC shell, it was shown that there was no bacteriophage activity. This effect may be related to the destruction of the shell by a proteinase with the subsequent release of monomers and dimers of the polyarginine shell, which may be the cause of bacteriophage inactivation [56,57]. 

PMCs (DS/PArg)_3_, destroyed by proteolytic enzymes, were incubated with bacteriophages to confirm this hypothesis. Usually, *E. coli* is present in the human intestine and can affect the process of phage decapsulation; arginine that forms as a result of the hydrolysis of the polyelectrolyte may be absorbed by the bacterium as a nutrient substrate, and it is possible to prevent bacteriophage inactivation [58]. For this reason, *E. coli* cells were incubated with destroyed microcapsules and bacteriophages. As can be seen from Table 2, the activity of bacteriophages is still present but significantly lower than that of free bacteriophages [59].

In the next stage of the work, we encapsulated bacteriophages in PMCs, but before plating it on nutrient media, we incubated it with *E. coli* and with proteinase P from Streptomyces griseus. In Table 1, the activity of bacteriophages is absent. These results agree with data from Table 2. This allows us to conclude that the polyarginine–dextran sulfate complex affects phage activity.

The effect of the polyelectrolyte complex (dextran sulfate–arginine, DS-Arg) on the activity of the bacteriophage can be electrostatic in nature; i.e., the polyelectrolyte complex can bind to the nonuniformly charged surface of the protein capsid. One of the ways to neutralize this effect is to create an electrostatic “coat”, or to bind arginine monomers with SO_4_^2−^ ions. For this purpose, the destroyed PMCs were incubated with magnesium sulfate (MgSO_4_) and sodium chloride, the final concentrations of which were 15 mM and 140 mM, and then bacteriophages were added. As can be seen from Table 2, the high ionic strength of the solution slightly prevented the inhibition of the bacteriophage, while magnesium sulfate acted more efficiently, as the number of surviving phages was 3.3%.

Subsequently, bacteriophages were encapsulated in polyelectrolyte microcapsules with a dissolved and undissolved CaCO_3_ core. The resulting PMCs were incubated in a solution of magnesium sulfate (MgSO_4_), the final concentration of which was 200 mM, before the destruction of the membrane by proteinase. From Table 1, it can be seen that magnesium sulfate did not prevent phage inhibition. To verify this result, we used a spectrophotometric method. The results agree with the data in Table 2.

**Figure 7:**
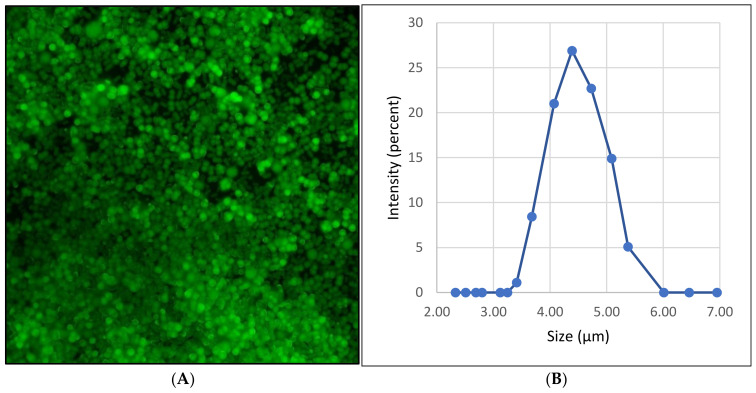
Image and size distribution of CaCO_3_ spherulites with immobilized bacteriophages. (**A**) Fluorescence microscopy image CaCO_3_ spherulites with immobilized FITC-labeled bacteriophages; (**B**) size distribution by intensity of CaCO_3_ spherulites with immobilized bacteriophages.

Based on the results of the presented work, it was concluded that PMCs are not a suitable means of delivering bacteriophages. However, the problem of maintaining the activity of the bacteriophage in the acidic environment of the digestive system still remains. In work by Smith W. et al. [58], to increase the survival of the bacteriophage in the acidic environment of the stomach, its pH was changed by using a suspension of calcium carbonate. However, the acidic environment of the stomach is necessary for the normal functioning of the gastrointestinal tract; therefore, we propose including bacteriophages in CaCO_3_ spherulites. Thus, the acidic pH is neutralized locally, creating favorable conditions for the bacteriophage but not having a critical effect on the overall pH of the stomach. An image of immobilized bacteriophages (Figure 7A) and the size distribution (Figure 7B) of CaCO_3_ spherulites are shown in Figure 6. The size of resulting CaCO_3_ particles with immobilized bacteriophage is 4.5 ± 1 µm.

**Figure 8:**
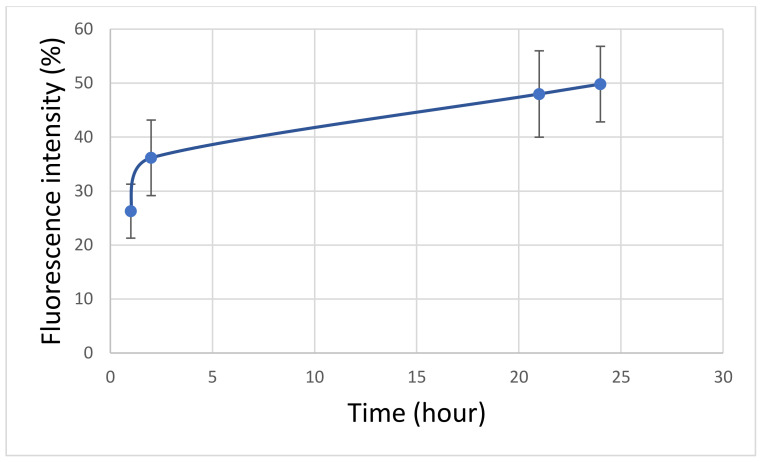
Bacteriophage release from CaCO_3_ spherulites depending on the incubation time.

After immobilization of the bacteriophages in CaCO_3_, the amount of bacteriophage released from CaCO_3_ depending on the incubation time was determined. To that end, FITC-labeled bacteriophages were immobilized in the CaCO_3_ cores and incubated in an aqueous solution at 37 °C. Figure 8 shows that the release of bacteriophages from CaCO_3_ cores was 26% at the first hour of incubation (100%—the total labeled bacteriophage immobilized in the CaCO_3_ cores). This effect may be due to the fact that the bacteriophage, which is fixed on the surface, is released into the solution at the beginning of incubation. The release of bacteriophages from CaCO_3_ cores after the second hour of incubation is insignificant and does not exceed 2%. Thus, it can be concluded that a significant proportion of bacteriophages leave CaCO_3_ particles in the first hour of incubation. 

**Figure 9:**
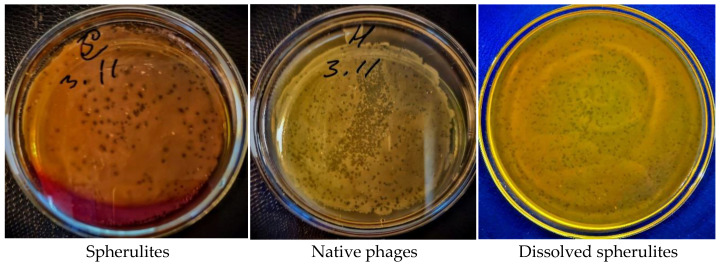
Influence of bacteriophages immobilized in CaCO_3_ cores on the activity of phages.

As can be seen from Figure 9, bacteriophages immobilized in CaCO_3_ cores have visible activity (98 plaques); the number of clear zones is less than in free phages (562 plaques), but the diameter of the clear zones in the case of immobilized phages in CaCO_3_ cores is much larger (d = 4–5 mm) than those created by free bacteriophages (d = 0.5–1 mm). This effect may be associated with the presence of active bacteriophages on the surface of CaCO_3_ particles. With the destruction of CaCO_3_, phages are observed to have high activity, which is equal to the activity of free bacteriophages (615 plaques).

As a result of this work, we recommend using CaCO_3_ particles as containers for bacteriophage delivery, because CaCO_3_ allows us to neutralize acidic local medium and to create favorable conditions for bacteriophages without critically affecting the pH of the stomach and digestion system. Furthermore, CaCO_3_ particles have good biocompatibility [59] and can be safely used for biomedical applications [60,61], and CaCO_3_ particles are cleared from the body due to digestion, phase transformation or dissolution [61].

## 4. Conclusions

The effect of pH 9.02 (pH of the medium during spherulite formation) on the activity of bacteriophages and *E. coli* was tested. It was found that the bacteriophages and *E. coli* retained their activity at a pH of 9.02.

Subsequently, the effect of proteolytic enzymes on the destruction of the polyelectrolyte microcapsule envelope was studied: trypsin, proteinase P from *Streptomyces griseus* (SIGMA), trypsin from bovine pancreas, protease from a strain of *B. subtilis*, and protease from *Streptomyces griseus* (SERVA). It was revealed that only protease from *Streptomyces griseus* was able to lyse the membrane of the microcapsule. Then, the effect of this proteolytic enzyme on the activity of the bacteriophage was studied, and it was shown that the viability of phages decreased in the second hour of incubation and then gradually decreased to a complete loss of activity after 16 h.

The bacteriophages were encapsulated into polyelectrolyte microcapsules. The incubation of encapsulated bacteriophages in an aqueous solution at 37 °C did not affect phage release. The activity of both encapsulated bacteriophages and free phages was studied in the presence of destroyed PMCs. It was found that despite the presence of active forms of the bacteriophage in the medium containing destroyed capsules and sulfate ions, it is impossible to use microcapsules as delivery vehicles for bacteriophages, since in all cases, the bacteriophages were inactive.

The final stage of the work was the development of a bacteriophage delivery vehicle inside a CaCO_3_ carrier. The release of bacteriophages from CaCO_3_ particles was investigated. It was found that 26% of bacteriophages were released from CaCO_3_ particles in the first hour of incubation. In addition, the results show that the CaCO_3_ particle can be used as a container for a bacteriophage since the phage retained its activity not only after the dissolution of the particle but also while inside of it.

Thus, we propose a new method for the delivery of bacteriophages using CaCO_3_ particles, which protects the bacteriophage when passing through the acidic environment of the stomach.

## Figures and Tables

**Table 1:** Activity of encapsulated bacteriophages in biodegradable polyelectrolyte microcapsules.

Capsule Type		Number of Plaques
Dissolved core	(DS/PArg)_3_	0
Undissolved core	(DS/PArg)_3_	3 ± 1
Dissolved core	(DS/PArg)_3_ + PDADMAC	0
Dissolved core + added *E. coli*	(DS/PArg)_3_	0
Undissolved core + added MgSO_4_	(DS/PArg)_3_	0
Dissolved core + added MgSO_4_	(DS/PArg)_3_	0
	Phages	65 ± 3

**Table 2:** Influence of dissolved capsules on phage.

Type of Dissolved Capsule	Number of Plaques
(DS/PArg)_3_	6 ± 1
(DS/PArg)_3_ + *E. coli*	9 ± 2
(DS/PArg)_3_ + 140 mM NaCl	4 ± 1
(DS/PArg)_3_ + 15 mM MgSO_4_	19 ± 2
Phages	642 ± 23
